# Supplementary material for: Steam Gasification of Torrefied/Carbonized Wheat Straw for H2-Enriched Syngas Production and Tar Reduction
Source: Int J Environ Res Public Health. 2022 Aug 23;19(17):10475. doi: 10.3390/ijerph191710475 (PMC9518206; doi:10.3390/ijerph191710475)
Supplement: Supplementary file 1 [file ijerph-19-10475-s001.zip › ijerph-1849532-supplementary.pdf]

Steam Gasification of Torrefied/Carbonized Wheat Straw for H_2-Enriched Syngas Production and Tar Reduction

Kejie Wang †, Ge Kong †, Guanyu Zhang, Xin Zhang, Lujia Han and Xuesong Zhang *

Engineering Laboratory for AgroBiomass Recycling & Valorizing, College of Engineering, China Agricultural University, Beijing 100083, China

* Correspondence: xszhang@cau.edu.cn

† These authors contributed equally to this work.

Supplementary

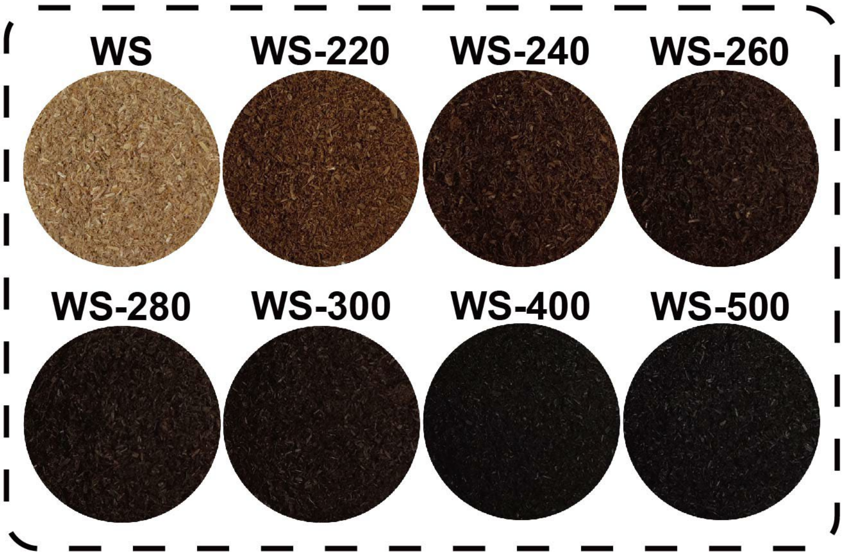

Figure S1 Photographs of raw WS and WS treated at various temperatures.

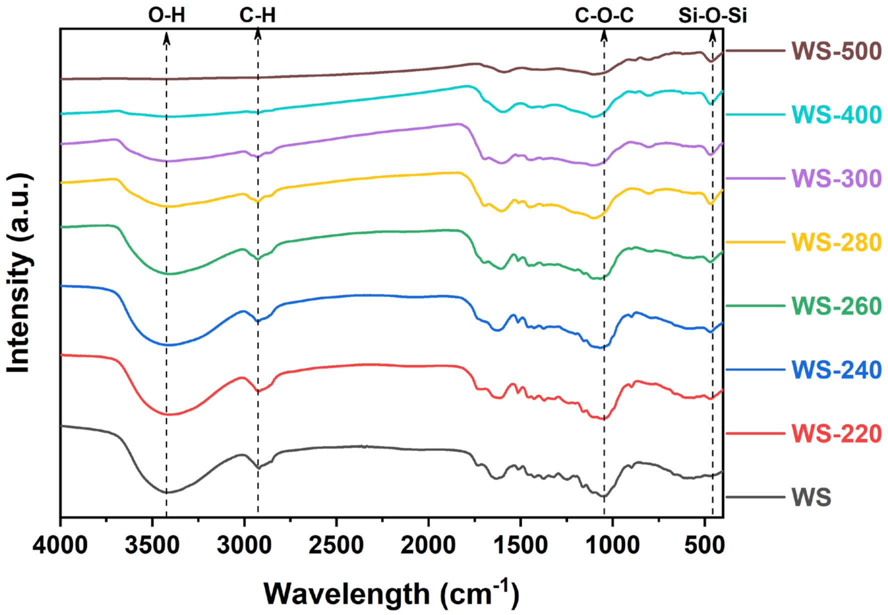

Figure S2 FTIR spectra of raw WS and WS treated at varying temperatures.
